# Effect of telehealth‐based versus in‐person nutritional and exercise intervention on type II diabetes mellitus improvement and efficiency of human resources utilization in patients with obesity

**DOI:** 10.1002/osp4.667

**Published:** 2023-04-28

**Authors:** Shruthi Rajkumar, Elana Davidson, Michael Bell, Christina Reardon, Abby Lapolla, Maria Michelakis, Yannis Raftopoulos

**Affiliations:** ^1^ Department of Weight Management Program Institution of Holyoke Medical Center Holyoke Massachusetts USA

**Keywords:** diabetes mellitus, glycated hemoglobin A, obesity, telemedicine, weight reduction programs

## Abstract

**Aims:**

Telehealth became a patient necessity during the COVID pandemic and evolved into a patient preference in the post‐COVID era. This study compared the % total body weight loss (%TBWL), HbA1c reduction, and resource utilization among patients with obesity and diabetes who participated in lifestyle interventions with or without telehealth.

**Methods:**

A total of 150 patients with obesity and diabetes who were followed every 4–6 weeks either in‐person (*n* = 83) or via telehealth (*n* = 67), were included. All patients were provided with an individualized nutritional plan that included a weight‐based daily protein intake from protein supplements and food, an activity/sleep schedule‐based meal times, and an aerobic exercise goal of a 2000‐calorie burn/week, customized to patient's preferences, physical abilities, and comorbidities. The goal was to lose 10%TBWL. Telehealth‐based follow‐up required transmission via texting of weekly body composition measurements and any blood glucose levels below 100 mg/dl for medication adjustments. Weight, BMI, %TBWL, HbA1c (%), and medication effect score (MES) were compared. Patient no‐show rates, number of visits, program duration, and drop‐out rate were used to assess resource utilization based on cumulative staff and provider time spent (CSPTS), provider lost time (PLT) and patient spent time (PST).

**Results:**

Mean age was 47.2 ± 10.6 years and 74.6% were women. Mean Body Mass Index (BMI) decreased from 44.1 ± 7.7–39.7 ± 6.7 kg/m^2^ (*p* < 0.0001). Mean program duration was 189.4 ± 169.3 days. An HbA1c% unit decline of 1.3 ± 1.5 was achieved with a 10.1 ± 5.1%TBWL. Diabetes was cured in 16% (24/150) of patients. %TBWL was similar in regards to telehealth or in‐person appointments (10.6% ± 5.1 vs. 9.6% ± 4.9, *p* = 0.14). Age, initial BMI, MES, %TBWL, and baseline HbA1c had a significant independent effect on HbA1c reduction (*p* < 0.0001). Program duration was longer for in‐person follow‐up (213.8 ± 194 vs. 159.3 ± 127, *p* = 0.019). The mean annual telehealth and in‐person no‐show rates were 2.7% and 11.2%, respectively (*p* < 0.0001). Mean number of visits (5.7 ± 3.0 vs. 8.6 ± 5.1) and drop‐out rates (16.49% vs. 25.83%) were lower in telehealth group (*p* < 0.0001). The CSPTS (440.4 ± 267.5 min vs. 200.6 ± 110.8 min), PLT (28.9 ± 17.5 min vs. 3.1 ± 1.6 min), and PST (1033 ± 628 min vs. 113.7 ± 61.4 min) were significantly longer (*p* < 0.0001) for the in‐person group.

**Conclusions:**

Telehealth offered comparable %TBWL and HbA1c decline as in‐person follow‐up, but with a shorter follow‐up, fewer appointments, and no‐shows. If improved resource utilization is validated by other studies, telehealth should become the standard of care for the management of obesity and diabetes.

## 1 INTRODUCTION

The impact of telehealth on the management of adults with obesity and T2DM has been explored in a few small pilot studies. The weight‐loss rates using the Diabetes Prevention Program (DPP) via videoconferencing were similar to those with in‐person visits.^1^ Telehealth lifestyle coaching has been shown to improve long‐term weight loss for people with obesity and T2DM compared to usual care.^2^, ^3^ A recent study suggested that the virtual format of the Why WAIT program was feasible and potentially as successful as the in‐person program in a small pilot study.^4^

Little is also known about the effect of telehealth on resource utilization. Arora et al reported a significant increase in prescription use during telehealth care but not specifically for the treatment of diabetes and obesity.^5^ Telehealth implementation in an adolescent bariatric surgery program led to an increase in attendance rates (83.1% vs. 70.5%) and a reduction in cancellation rates (9.9% vs. 22.5%) compared to in‐person appointments.^6^ Furthermore, conversion to a total telehealth approach for the preoperative bariatric multidisciplinary workup decreased the time to surgery and the number of total outpatient visits and time to surgery but no weight loss was achieved.^7^ In addition, a statistically non‐significant reduction in bariatric group‐visit no‐show rates (16.45% vs. 11.54%) has been reported after telehealth implementation in a bariatric surgery program, but correlation with weight loss outcomes or comorbidity improvement was not examined.^8^ The effect of telehealth on resource utilization specifically for the management of adults with obesity and diabetes and in conjunction with clinical outcomes has not been previously investigated.

The study hypothesized that telehealth would achieve similar weight loss and diabetes improvement compared to in‐person visits but with fewer resources.

## 2 MATERIALS AND METHODS

### 2.1 Study design

Between March 2016 and April 2022, a total of 150 patients with obesity (BMI ≥30 kg/m^2^) and T2DM were enrolled in the weight loss program either by referral from the Primary Care Provider or Endocrinologist, word of mouth or in response to online advertisements by the institution's marketing department. There was no specific fee assigned to the program except for direct billing to each patient's insurance plan for each office appointment and in‐person group class. Online classes were accessible without charge. Patients had either only in‐person visits between March 2016 and February 2020 or telehealth visits only since March 2020 when the COVID pandemic began. Since this was not a randomized study, sample size calculation and power analysis were not performed. Data were prospectively collected in an IRB‐approved registry. Only the data missing from the registry was searched retrospectively in the electronic medical record (EMR). As per American Diabetes Association, T2DM was defined as HbA1c greater than or equal to 6.5%.^9^ Patients with ages less than 18 years, patients who were considering pregnancy, patients with a history of bariatric surgery, and patients with type I diabetes mellitus were excluded from the study. The study was approved by the IRB and all patients provided informed consent to use the data for research purposes.

### 2.2 Weight loss program

It was an individualized multidisciplinary program aiming at 10% total body weight loss (TBWL). Patients of either the in‐person or telehealth group were scheduled to have a 1:1 appointment with the assigned provider every 4–6 weeks. The intervention was not based on a predetermined length but on a 10% TBWL goal or until the patient decided to drop out of the program. Because the intervention was not based on a predetermined length but on a specific weight loss goal, it was individualized until each patient met the goal. The key components of the program included nutrition, behavioral health, exercise, and nutritional classes.

#### 2.2.1 Nutrition

All patients followed individualized nutritional plans which were focused on protein amount and not calorie intake. The plans included a combination of protein shakes, bars, and one food‐based meal. The food‐based meal combined a lean protein source with a salad or vegetable. Emphasis was placed on teaching the patient to measure the food portions correctly as it was factored into the daily protein intake. Patients were allowed to replace half of the salad/vegetable portions with high carbohydrate choices such as rice, pasta, potatoes, bread, fruits, or desserts to improve long‐term adherence. The meal portions and the daily number of protein shakes and bars were not the same for all patients. The patients were not advised to use any specific brand of protein shakes or bars. The selection was based on each patient's protein intake needs and preferences. Powdered protein shakes were preferred, but premade shakes were also used per the patient's preference. The choice of milk used to mix the powdered shakes was calculated into the daily protein intake goal. The number of eating episodes, meal and shake portions, and the time of intake for each eating episode was not standard; instead, were adjusted to each patient's daily schedule and sleep habits. The plan could be different from day to day for the same patient depending on the schedule. The daily protein intake was based on the patient's current weight and was modified as the patient lost weight.^10^ Water intake was encouraged but no daily specific intake goals were set. Initial comprehensive blood tests and an abdominal ultrasound with elastography were performed. Any vitamin or mineral deficiencies were treated, and subsequent blood levels were checked to ensure adequate correction.

#### 2.2.2 Behavioral health

A behavioral health specialist evaluated all patients. Patients with active untreated depression or anxiety, binge eating behaviors, or recent addictive history were engaged in further individual therapy.

#### 2.2.3 Exercise

Patients were given specific directions for aerobic and resistance exercises. Exercise programs were customized based on each patient's physical conditioning, age, and musculoskeletal limitations. The type of recommended aerobic exercise (walking, treadmill, bike, elliptical, or rowing), as well as at home or a fitness center, was based on equipment availability and each patient's characteristics. The patients were encouraged to purchase a treadmill, stationary bike, or elliptical for home use. The program was based on a specific weekly calorie‐burning goal of 2000 calories. To achieve this goal, each patient was educated to break down the weekly calorie goal into a specific calorie goal for each workout based on the number of days per week each patient intended to exercise. Each patient was also instructed to break down each workout calorie goal into more than one workout session per day if the patient experienced musculoskeletal pain or was too deconditioned to complete the calorie goal in one session. Exercise intensity was adjusted based on each patient's weight loss and length of participation.

#### 2.2.4 Nutritional classes

These were aimed to educate patients on important nutritional, behavioral, and exercise topics and focused on teaching new skills, including cooking demonstrations, exercise, and breathing techniques. In‐person classes were conducted by a dietitian, physician assistant, and physical therapist aimed to provide support and create a sense of community among the patients. The telehealth group received a link either by text or email to complete these classes online. Both groups had to complete a quiz after each class and those were reviewed by the dietitian.

### 2.3 Telehealth

The program offered the option of telehealth appointments via videoconferencing or telephone. Additionally, patients were required to purchase a smartphone app‐based body composition analyzer, a glucometer, and a blood pressure monitor. Every effort was made that the necessary equipment was prescribed through each patient's insurance plan. The patients were instructed to send the weight measurements weekly via text message. For patients who were either unable to purchase the necessary equipment or had difficulty transmitting the required data to the provider, the staff provided weight checks in the office in between the scheduled appointments or assistance with the app‐based body composition analyzer with the medical assistants. Weight checks in the office were not counted as appointments. Patients were also asked to electronically send the blood glucose and blood pressure measurements for medication dose adjustments. The frequency of assessments varied daily to weekly based on each patient's needs and was adjusted over time. Patients were instructed to text to the assigned provider any blood glucose <100 mg/dl or >150 mg/dl and blood pressure >130/80 mmHg or <120/70 mmHg. The patients received feedback within 24 h, regarding the weight loss progress, and any adjustments that had to be made to the nutritional or exercise plan and antidiabetic or antihypertensive medications. All patients assigned to the telehealth group were able to participate based on the above pathway.

### 2.4 In‐person appointments

In‐person appointments were scheduled at the office. Patients were required to purchase a glucometer and a blood pressure monitor. A body composition scale was not required as the patients were scheduled for in‐person appointments and were weighed in the body composition scale at the office. Patients were encouraged to come to the office in between the scheduled appointments for weight checks with the medical assistants. Patients were instructed to call the office for blood glucose <100 mg/dl or >150 mg/dl and blood pressure >130/80 mmHg or <120/70 mmHg.

### 2.5 Data collection

The following data were collected: baseline demographics, weight (kg), BMI, body composition, antidiabetic medications with doses, program duration, HbA1c, fasting blood glucose, blood insulin levels, lipid panel, liver function tests, C‐reactive protein, blood urea nitrogen and creatinine levels. Medication Effect Scores (MES) were used to quantify the intensity of the antidiabetic regimen calculated and were calculated based on the formula: MES = (actual dose/maximum dose) x adjustment factor, which was the expected decrease in HbA1c for the medication.^11^ A higher MES score indicated increased antidiabetic medication use.^12^

### 2.6 Assessment of resource utilization

Program duration, the total number of appointments per patient, and no‐show rates were used to assess resource utilization. Program duration was defined as the length (days) of each patient's active participation from the initial appointment until the last appointment. The total number of appointments was defined as the sum of all in‐person or telehealth (telephone or video‐assisted) appointments that were booked at the providers' office schedule and were completed.

Blood glucose, blood pressure, and body composition assessments were not considered as appointments and were assessed separately as described below. Each patient's no‐show rate was calculated as the percentage of non‐completed appointments (without prior cancellation or cancellation less than 24 h from the time of appointment) in relationship to the total number of scheduled appointments. Booked appointments that were canceled by the patient at least 24 h before the actual appointment were not included in the calculation of the no‐show rate as the institution's EMR allows removal from the provider's schedule.

Cumulative staff and provider time spent, provider time lost because of no‐shows, and patient time spent for the appointments were calculated. An average time of 20 min per telehealth and 30 min per in‐person appointment was used for all calculations. Cumulative staff and provider time spent was the sum of text messaging (2.5 min on average per week of active participation for telehealth patients only), appointment time, patient rooming time (0 for telehealth and 15 min for in‐person appointments), patient appointment scheduling (5 min per appointment) and patient appointment re‐scheduling (10 min per no‐show appointment). Medical assistant time utilization for weight checks in between appointments was not included in the assessment because this occurred in both groups evenly and the time allocated was minimal and similar for the two groups. Provider lost time was calculated per each no‐show appointment. Patient time was calculated as the sum of average appointment time plus time spent commuting to the office (60 min, only for in‐person appointments) plus wait time (average for telehealth: 5 min and for in‐person visits: 30 min). Program drop‐out rates were calculated as the percentage of patients who were active less than 30 days from the initial appointment in relation to the total number of patients seen for the same period (in‐person: 3/2016 to 2/2020 and telehealth: 3/2020 to 4/2022).

### 2.7 Statistical analysis

Descriptive statistics with mean, standard deviations (SD), and *t*‐tests were used for continuous variables, and Fisher's exact test for categorical variables. Regression analysis was performed to determine if %TBWL, program duration, initial BMI, age, gender, telehealth group, MES, and initial HbA1c had an independent effect on HbA1c reduction. Two additional regression models were used to assess the independent effect of age, gender, initial BMI, %TBWL, telehealth group, and either program duration or the number of appointments on either the number of appointments or program duration respectively. Multicollinearity was assessed and was not a problem for any of the regression analysis models. Statistical significance was defined as *p* < 0.05. Analysis was performed using GraphPad InStat statistical software (version 3.10, La Jolla, CA).

## 3 RESULTS

### 3.1 Demographics

A total of 150 patients with a mean age of 47.2 years, BMI of 44.1 kg/m^2^, and 74.6% of women, who had either in‐person (*N* = 83) or telehealth (*N* = 67) appointments were included in the study (Table 1). At an average program duration of 189.4 ± 169.3 days, the mean weight reduced from 117.6 ± 22.7 kg at baseline to 105.6 ± 19.7 kg (*p* < 0.001), resulting in a mean weight loss of 12 kg and %TBWL of 10.1 ± 5.1%. The mean BMI dropped from 44.1 ± 7.7 kg/m^2^ to 39.7 ± 6.7 kg/m^2^ (*p* < 0.001), and the mean fat percentage decreased from 43.4 ± 5.5% to 41.9 ± 9.0% (*p* = 0.0046). A statistically significant reduction in systolic and diastolic blood pressure from 138.5 ± 16.0–132.0 ± 13.7 mmHg (*p* = 0.0015) and from 73.7 ± 10.7–70.4 ± 9.5 mmHg (*p* = 0.022), respectively was noted (Table 1).

**TABLE 1:** Demographics and body weight and composition at baseline and last visit.

Parameters	Baseline	Last visit	*p‐*value
Mean age (years)	47.2 ± 10.6		
Gender			
Women	112 (74.6%)		
Men	38 (25.4%)		
Mean weight (kg)	117.6 ± 22.7	105.6 ± 19.7	<0.0001
Mean BMI (kg/m^2^)	44.1 ± 7.7	39.7 ± 6.7	<0.0001
Mean body fat percentage (%)	43.4 ± 5.5	41.9 ± 9.0	0.0046
Mean %TBWL (%)	10.1 ± 5.1		
Mean duration of the program (days)	189.4 ± 169.3		
Mean SBP (mmHg)	138.5 ± 16.0	132.0 ± 13.7	0.0015
Mean DBP (mmHg)	73.7 ± 10.7	70.4 ± 9.5	0.0220

*Note*: Values are presented as mean ± SD unless otherwise specified.

Abbreviations: %TBWL, Total Body Weight Loss Percentage; DBP, Diastolic Blood Pressure; SBP, Systolic Blood Pressure.

### 3.2 Glycemic parameters and medications

The mean HbA1c% reduced from 8.1 ± 1.4% at baseline to 6.8 ± 1.5% (*p* < 0.001) at program completion resulting in a mean HbA1c% unit reduction of 1.3%. The percentage of patients with HbA1c <7% increased from 22.6% (34/150) at baseline to 66.6% (100/150) (*p* < 0.001) at the last appointment. The mean fasting serum glucose reduced from 164 ± 62.6 mg/dl to 130.0 ± 54.2 mg/dl (*p* < 0.001), and the serum insulin levels decreased from 32 ± 48.7 uU/ml to 27.7 ± 61.6 uU/ml (*p* < 0.001).

The mean number of antidiabetic drugs decreased from 1.8 ± 1.2 at baseline to 1.5 ± 1.1 (*p* < 0.036) at program completion. There was a statistically non‐significant reduction of patients treated with insulin, regardless of dosage from 43.3% (65/150) at baseline to 37.3% (56/150) at program completion. Long‐acting insulin doses significantly reduced from baseline (44.3 ± 38.7 to 30.9 ± 35.9 units, *p* = 0.035) (Table 2). At the end of the program, 49.3% (74/150) had HbA1c < 6.5% regardless of the medication status, and 16% (24/150) had HbA1c < 6.5% without antidiabetic medications. Changes in other metabolic parameters are summarized in Table 3.

**TABLE 2:** Glycemic parameters and antidiabetic medications at baseline and last visit.

Parameters	Baseline	Last visit	*p*‐value
Mean HbA1c (%)	8.1 ± 1.4	6.8 ± 1.5	<0.001
Mean fasting blood glucose (mg/dl)	164.0 ± 62.6	130.0 ± 54.2	<0.001
Mean blood insulin levels (uU/ml)	32.0 ± 48.7	27.7 ± 61.6	<0.001
Mean number of T2DM medications	1.8 ± 1.2	1.5 ± 1.1	<0.036
Mean medication effect Score (MES)	1.7 ± 1.7	1.5 ± 1.6	0.2553
Patients taking insulin	65 (43.3%)	56 (37.3%)	0.4910
Long‐acting insulin dose (units)	44.3 ± 38.7	30.9 ± 35.9	0.0350
Patients taking T2DM medications	127 (84.6%)	121 (80.6%)	0.4459
Patients with HbA1c < 7	34 (22.6%)	100 (66.6%)	<0.001

*Note*: Values are presented as mean ± SD unless otherwise specified.

Abbreviation: T2DM, Type II Diabetes Mellitus.

**TABLE 3:** Other Metabolic parameters at baseline and the end of the program.

Parameters	Baseline	Last visit	*p*‐value
Total serum protein (g/dl)	7.27 ± 0.47	7.18 ± 0.04	0.1636
Serum albumin (g/dl)	4.51 ± 2.70	4.25 ± 0.27	0.5741
Serum creatinine (mg/dl)	0.90 ± 0.28	0.89 ± 0.26	0.4772
Serum C‐reactive protein (mg/dl)	1.5 ± 1.2	1.3 ± 1.0	0.1101
Total Cholesterol (mg/dl)	169.6 ± 43.0	167.8 ± 44.8	0.7480
Serum triglyceride (mg/dl)	158.0 ± 84.5	131.7 ± 57.4	0.0030
Serum LDL (mg/dl)	98.8 ± 38.2	103.0 ± 39.4	0.3766
Serum HDL (mg/dl)	40.2 ± 9.8	39.4 ± 9.1	0.5103

*Note*: Values are presented as mean ± SD unless otherwise specified.

Abbreviations: HDL, High‐density lipoprotein; LDL, Low‐density lipoprotein.

### 3.3 Telehealth versus in‐person appointment groups

There was no significant difference in mean HbA1c reduction or %TBWL between the in‐person and telehealth groups. Compared to the telehealth, the in‐person group had a significantly longer mean program duration (213.8 ± 194.0 vs. 159.3 ± 127.0, *p* = 0.019), a higher mean annual no‐show rate (11.2% vs. 2.7%, (*p* < 0.001) and more appointments (8.6 ± 5.1 vs.5.7 ± 3.0 *p* < 0.001) per patient (Table 4).

**TABLE 4:** Comparison between In‐Person visit group and Telehealth group.

Mean	In‐person visit group (*n* = 83)	Telehealth group (*n* = 67)	*p*‐value
Age (years)	49.3 ± 10.5	44.8 ± 10.2	0.0052
Female (%)	77.1	71.6	0.4569
Weight reduction (kg)	11.4 ± 7.3	12.6 ± 7.9	0.1531
BMI reduction (kg/m^2^)	4.2 ± 2.4	4.8 ± 2.9	0.1301
Baseline HbA1c (%)	7.9 ± 1.0	8.3 ± 1.8	0.7222
HbA1c difference (%)	1.1 ± 1.0	1.3 ± 1.9	0.9830
Baseline MES	1.7 ± 1.7	1.8 ± 1.7	0.6167
%TBWL	9.6 ± 4.9	10.6 ± 5.1	0.1400
Duration of program (days)	213.8 ± 194.0	159.3 ± 127.0	0.0190
Annual mean no‐show rates (%)	11.2	2.7	<0.001
Program drop‐out rates (%)	193/747 (25.83%)	190/1152 (16.49%)	<0.0001
Number of visits/patient	8.6 ± 5.1	5.7 ± 3.0	<0.001
Cumulative staff and provider time spent (min)	440.4 ± 267.5	200.6 ± 110.8	<0.0001
Provider lost time (min)	28.9 ± 17.5	3.1 ± 1.6	<0.0001
Patient spent time (min)	1033.0 ± 628.0	113.7 ± 61.4	<0.0001

*Note*: Values are presented as mean ± SD unless otherwise specified.

Abbreviations: %TBWL, Total Body Weight Loss Percentage; BMI, Body Mass Index; MES, Medication Effect Score.

On regression analysis, baseline HbA1c (95% CI: 0.4343–0.7029, *r* = 0.4980, *p* < 0.0001), MES (95% CI: −0.2557 to −0.03161, *r* = −0.0990, *p* = 0.0131), initial BMI (95% CI: 0.002065–0.05440 *r* = 0.2444, *p* = 0.0362), age (95% CI: 0.001213–0.03947, *r* = 0.0157, *p* = 0.0389) and %TBWL (95% CI: 0.06717–0.1440, *r* = 0.3691, *p* < 0.0001) had a significant and independent effect on HbA1c reduction (*R* ^2^ = 44.96%, *p* < 0.0001).

The cumulative staff and provider time spent (440.4 ± 267.5 min vs. 200.6 ± 110.8 min, *p* < 0.0001), provider lost time (28.9 ± 17.5 min vs. 3.1 ± 1.6 min, *p* < 0.0001) and patient time spent (1033 ± 628 min vs. 113.7 ± 61.4 min, *p* < 0.0001) were significantly longer at the in‐person group. The program drop‐out rate significantly reduced (*p* < 0.001) when telehealth was implemented (Table 4). On regression analysis, program duration (95% CI: 0.01504–0.02136, *r* = 0.6719, *p* < 0.0001), telehealth (95% CI: −3.162 to −1.003, *r* = −0.3158, *p* = 0.0002) and %TBWL (95% CI: 0 0.06402–0.2742, *r* = 0.1136, *p* = 0.002) had a significant and independent effect on the total number of appointments (*R* ^2^ = 54.05%, *p* < 0.0001). Only the total number of appointments (95% CI: 21.443–30.467, *r* = 0.6719, *p* < 0.0001), %TBWL (95% CI: −9.293 to −1.267, *r* = −0.0438, *p* = 0.0109) and initial BMI (95% CI: 1.006–6.336, *r* = 0.1053, *p* = 0.0078), but not telehealth, had an independent effect on program duration (*R* ^2^ = 51.12%, *p* < 0.0001).

## 4 DISCUSSION

The mean reduction of 1.3% in %HbA1c unit and a mean %TBWL of 10.1 ± 5.1% in this study, over an average period of 27 weeks were comparable with the best‐published results. Quimby KR et al. implemented a very low‐calorie diet (LCD) (VLCD of 840 cal/day) for 12 weeks, which led to a rapid weight loss of 6.3%TBWL and a 1.1% decline in %HbA1c.^13^ A low‐carbohydrate diet and a low‐fat diet of approximately 1800 cal/day in conjunction with an exercise plan achieved a %TBWL of 5.8% and 1.4% and an HbA1c reduction of 1.7% and 0.9%, respectively.^14^ Bynoe K et al. achieved a 10.6% TBWL and a 0.9% HbA1c reduction with a LCD (760 cal/day) for 8 weeks and a 4‐week transition to food.^15^ Similarly, a %TBWL and %HbA1c reduction of 9.8% and 0.89% respectively were reported by the 12‐week Why Wait multidisciplinary program of the Joslin Diabetes Center.^16^

Although calories were inevitably restricted with the implementation of this study's nutritional protocol posing similarities with VLCDs and LCDs, there are also distinct differences: (1) compared to VLCDs and LCDs, calorie counting was not the main objective and there were no daily calorie intake goals; (2) since the nutritional plan included a food‐based meal and replacement of half of the salad portion with carbohydrates was allowed, the daily calorie intake was more than that of VLCDs, defined as 800 calories or less than 50% of the patient's basic metabolic rate^17^; (3) the daily protein intake goal was much higher than what is recommended in VLCDs and LCDs and was nearly 1 g/kg of actual body weight and not 0.8–1.5 g/kg of ideal body weight^17^; (4) the potentially prohibitive high cost of such nutritional plans for patients with low socioeconomic status reported by others was not observed in this study.^17^ Even though this program serves a population with multiple racial disparities (59% people of color, a median income of $42,000, 16% living in poverty, 42.6% speaking a language other than English, and 78% without a bachelor's degree),^18^ the drop‐out rate observed was not higher than previously reported (22%) and reduced substantially with telehealth implementation.^19^

Other than constipation, which was easily managed by conventional treatments and occurred primarily in patients who had it as a pre‐existing condition, this nutritional plan was very safe. Side effects frequently reported with VLCDs, such as cholelithiasis, hair loss, fatigue, electrolyte abnormalities, or muscle cramps were not observed.^17^ Headache was observed in patients who reduced caffeine intake concurrently. Cold intolerance was not observed as this program includes an individualized aerobic exercise plan which offset the impact of calorie reduction. Dizziness was reported occasionally by patients, who were on antihypertensive medications and did not report any blood pressure below 120/70 mmHg promptly, but resolved quickly after medication adjustment.

On regression analysis, this study demonstrated that a greater HbA1c reduction had significant independent linear relationships with a greater %TBWL, older age, higher baseline HbA1c, and BMI, and an inverse relationship with baseline MES. Of these factors, the only one that can be controlled is weight loss. Therefore, no matter how effective a program is, weight loss should not be compromised when different approaches, such as telehealth versus in‐person appointments are compared in delivering diabetic care more effectively or efficiently. This study demonstrated that telehealth offered equal benefits in weight loss and glycemic management as in‐person appointments.

A study of 894 patients with a BMI greater than 25 kg/m^2^ and one or more risk factors of cardiovascular disease or diabetes utilizing the DPP lifestyle intervention for 6 months found that patients assigned to telehealth or on‐site participation achieved a comparable weight loss of 5.5 kg.^1^ Although the mentioned study reinforces the observation of similar weight loss between telehealth and on‐site interventions, it did not focus on patients with obesity and medically treated diabetes, did not report glycemic control and diabetes improvement, and achieved less than half the weight loss that was achieved in this study. Christensen et all, also reported a similar weight loss and HbA1c reduction among patients who received telehealth lifestyle coaching and usual care for 24 months.^2^ Although in contrast to the present study's protocol, the control group in Christensen et al did not receive the same care as the telehealth group, and the weight loss (3.9 vs. 12.6 kg) and HbA1c reduction (0.4% vs. 1.3%) were lower compared to that in this study at 6 months. Yin et al compared the management of patients with diabetes and BMI >25 kg/m^2^, with conventional biweekly in‐person follow‐up versus an app‐assisted telehealth approach and observed a similar HbA1c reduction between the two groups and a greater BMI reduction in favor of the telehealth group, but overall lower than this study (3.77 kg/m^2^ vs. 4.76 kg/m^2^).^3^ In contrast to the current study, however, the two groups did not receive the same frequency of follow‐up as patients in the telehealth group were followed 4 times per week whereas the control group was followed in‐person biweekly.^3^ Furthermore, a recent study suggested that a virtual format of the Why WAIT program produced comparable weight loss (7.4 vs. 6.9 kg) and HbA1c reduction (1.03% vs. 1%) as in‐person appointments.^4^

The current study demonstrates a significant benefit of the telehealth approach in terms of resource utilization compared to the in‐person approach while maintaining a comparable weight loss and HbA1c reduction. The cumulative time spent by staff and providers, the provider time lost by no‐shows, and the time patients utilized for the appointments were significantly lower in favor of the telehealth group. The significant reduction in the time spent by the patients for the appointments when the telehealth approach was used, probably played an important role in the reduction of no‐shows (11.2%–2.7%) and program drop‐out rates (25.83%–16.49%). In a recent study by Brown et al.,^8^ no‐show rates of initial and established visits of patients with severe obesity before bariatric surgery did not significantly improve after telehealth implementation in a bariatric surgery practice except for a small fraction of established visits with the bariatrician. The mentioned study, however, did not assess other important factors such as program duration, weight loss, or diabetes status and improvement and included pre‐bariatric and post‐bariatric surgery visits. In accordance with the results of the current study, the no‐show rate of telehealth visits in a primary and specialty care clinic during the COVID pandemic was significantly lower (7.5%) than both the no‐show rate of 36.1% for in‐office visits that occurred during the same period and a pre‐pandemic in‐office no‐show rate of 29.8%.^20^ Similarly, the no‐show rates reported in a weight management program were lower for telehealth (11.5%) than in‐person (15.8%) visits.^21^

On univariate analysis, this study demonstrated a significant reduction in program duration and the number of appointments when the telehealth approach was utilized. This was in agreement with a recent study by Mills et al which reported a significant reduction in time and the number of visits from the initial appointment to bariatric surgery when patients received telehealth‐based versus in‐person care. Mills et al, however, did not assess no‐show rates or achieved any weight loss.^7^ Every effort was made that both groups in the present study were scheduled to have the same appointment frequency to reduce any effect on program duration. This is supported by this study's regression analysis which did not identify telehealth as an independent factor influencing program duration, whereas initial BMI, %TBWL and the total number of appointments were independently related to program duration. In contrast, telehealth was an independent factor of a reduced number of total appointments required until program completion. As a result, the differences in resource utilization between the two groups were not linked to bias related to this study design but primarily due to the benefits that the telehealth approach offers, such as better compliance, feedback, and communication between patients and providers leading to more effective weight loss and diabetes improvement. The weekly remote reporting of body composition measurements and blood glucose and blood pressure monitoring likely played a significant role in the more effective use of available resources.

These findings open new possibilities for the efficient delivery of cost‐effective lifestyle interventions with the use of telehealth that should be considered even when in‐person visits are available. One such example that was not fully captured in this study's data but anecdotally observed in telehealth was the easier consolidation of providers' short schedule gaps due to no‐shows by rearranging the appointment times of all telehealth appointments for that day. This allowed the providers to complete the office responsibilities earlier and have more solid time to focus on research, clinical or administrative duties. This would be impossible if such gaps occurred between in‐person appointments as such visits are extremely difficult to be moved up or changed in the provider's schedule from the staff's experience. As a result, the provider may have multiple unproductive short time gaps between appointments due to no‐shows that do not allow any complex, time‐consuming, or focused activities to occur.

Every effort was made to provide a fair assessment of the telehealth approach by concurrently comparing clinical outcomes and resource utilization for the first time as both should be taken into consideration in all studies comparing the two approaches. In contrast to other studies, this study did not exclude patients with BMI greater than 40 kg/m^2^, extreme HbA1c values, diabetic complications, insulin use, or use of a specific number of antidiabetic medications, as the aim was to create an obesity and diabetes program in which telehealth could be effectively applied in real‐world circumstances without exceptions or exclusions.

This study is not without limitations. Firstly, since the definition and calculation of no‐show rates in the reviewed published literature were not consistent or not clearly defined, the intention was to report the published results and a direct comparison with this study's data should be made with caution. In this study, booked appointments that were canceled by the patient at least 24 h before the due time were not included in the calculation of the no‐show rate as the institution's EMR allows the appointment removal from the provider's schedule and replacement with other patients from the waitlist. Since this study focused on resource utilization, cancellations made at least 24 h before an appointment did not affect this metric and were not included. Anecdotally, however, cancellations within 24 h from appointment time were much more frequent in the in‐person group simply because it was more difficult to keep up fixed‐time on‐site appointments when last‐minute unexpected events tend to occur in patients' day‐to‐day lives. Additionally, the replacement of canceled telehealth appointments was much easier than the replacement of in‐person appointments. Secondly, the calculation of no‐show rates was made in the same way for both groups and therefore allowed for a fair comparison of the two groups. In addition, free access to protein supplements or exercise equipment was not provided. Patients were only given directions on what to purchase or use. This, however, did not seem to affect the study's outcomes, even though, as stated previously, the target population was extremely challenging. In addition, there were inconsistent data on the duration of diabetes, and hence, its role in glycemic control could not be assessed. To counter this, the MES were used to assess the severity of diabetes. Ethnicity plays a role in the susceptibility to glycation of hemoglobin (HbA1c).^15^ Although this study did not assess the impact of ethnicity on the HbA1c improvements, 61% of the population is non‐white, with a significant percentage of the population being of Hispanic origin. Lastly, follow‐up on these patients for a year after completion of the program was not possible, as a significant percentage underwent bariatric surgery. Consequently, classifying the patients based on diabetes remission, which requires being free of T2DM medications for a year, was not possible.

## 5 CONCLUSION

This study shows that individualized meal and exercise plans for patients with obesity and T2DM are effective in weight loss and HbA1c improvements. The glycemic control and weight loss via telehealth visits are as good as in‐person visits. Moreover, telehealth‐based follow‐up can potentially decrease the duration of the weight loss program, the number of visits, and no‐show rates.

## CONFLICTS OF INTEREST STATEMENT

All authors have no conflicts of interest to declare.
